# Integration of single-cell regulon atlas and multi-omics data for prognostic stratification and personalized treatment prediction in human lung adenocarcinoma

**DOI:** 10.1186/s12967-023-04331-z

**Published:** 2023-07-25

**Authors:** Yi Xiong, Yihao Zhang, Na Liu, Yueshuo Li, Hongwei Liu, Qi Yang, Yu Chen, Zhizhi Xia, Xin Chen, Siyi Wanggou, Xuejun Li

**Affiliations:** 1 Department of Neurosurgery, Xiangya Hospital, Central South University, Changsha, 410008 Hunan China; 2 Hunan International Scientific and Technological Cooperation Base of Brain Tumor Research, Xiangya Hospital, Central South University, Changsha, 410008 Hunan China; 3 Xiangya School of Medicine, Central South University, Changsha, 410013 China; 4 Postdoctoral Research Workstation, Xiangya Hospital, Central South University, Hunan, 410078 China; 5 Department of Pharmacology & Toxicology, University of Toronto, Toronto, ON M5S 1A8 Canada; 6 Songjiang Research Institute, Shanghai Songjiang District Central Hospital, Shanghai Jiao Tong University, School of Medicine, Shanghai, 201600 China

**Keywords:** LUAD, LPRI, Prognostic model, Transcriptional regulation, TCGA, Single cell RNA sequencing, TME, Chemotherapy and immunotherapy

## Abstract

Transcriptional programs are often dysregulated in cancers. A comprehensive investigation of potential regulons is critical to the understanding of tumorigeneses. We first constructed the regulatory networks from single-cell RNA sequencing data in human lung adenocarcinoma (LUAD). We next introduce LPRI (Lung Cancer Prognostic Regulon Index), a precision oncology framework to identify new biomarkers associated with prognosis by leveraging the single cell regulon atlas and bulk RNA sequencing or microarray datasets. We confirmed that LPRI could be a robust biomarker to guide prognosis stratification across lung adenocarcinoma cohorts. Finally, a multi-omics data analysis to characterize molecular alterations associated with LPRI was performed from The Cancer Genome Atlas (TCGA) dataset. Our study provides a comprehensive chart of regulons in LUAD. Additionally, LPRI will be used to help prognostic prediction and developing personalized treatment for future studies.

**Supplementary Information:**

The online version contains supplementary material available at 10.1186/s12967-023-04331-z.

## Background

Transcriptional program dysregulation is a hallmark of cancer. Transcriptional regulation plays a crucial role in the cell identity maintenance of cancer cells and cancer-associated cells. Transcription factors (TFs) function by recognizing and binding specific sequences to regulate gene expression. The combinations of TFs and their target genes can sometimes control gene expression which may determine cell identity. Cancer is a complex ecosystem that comprises many different cell types including malignant cells, immune cells, and stromal cells. Understanding how transcriptional programs control different cell states and cell types in the cancer ecosystem will provide opportunities for therapeutic benefits in cancer.

Lung cancer has been the focus of cancer research for many years. According to the GLOBOCAN 2020 estimation, lung cancer is a leading cause of cancer death worldwide [1]. Non-small cell lung cancer (NSCLC) patients account for nearly 85% of all lung cancer cases, and almost 50% of them suffer from lung adenocarcinoma (LUAD) [2]. Lung cancer is characterized by pronounced heterogeneity. In recent years, with the advances in genomics, the study from The Cancer Genome Atlas Research Network (TCGA) has characterized the major subtypes in the transcriptome and genome in LUAD [3]. Recently, single-cell RNA sequencing (scRNA-seq) has been used to assess transcriptional similarities and differences within a population of cells in cancer. Kim et al. used scRNA-seq to establish the atlas of primary and metastatic LUAD from human patients [4]. Jacks and colleagues analyzed the single-cell epigenome in a mouse model and found changes in epigenomic states from normal cells to different states of malignant cells with cancer progression, which was controlled by key transcription factors [5]. Up until now, the underlying gene regulatory networks (GRNs) at the single cell resolution for human LUAD remain unclear. In this study, we sought to characterize the GRNs in LUAD from scRNA-seq. Here we used the *SCENIC* pipeline to construct the GRNs from the published dataset (GSE131907) [4, 6]. The *SCENIC* algorithm infers the TFs and their potential target genes, which are jointly named a regulon. Next, we identified the regulons that were associated with different states of malignant cells. Although previous studies established the prognostic model in NSCLC with different biological insights such as immune-related genes [7], RNA splicing [8], a robust gene signature that considers to avoid the inherent technical biases across different sequencing platforms to predict prognosis is warranted. Additionally, the exploration and the identification of novel biomarkers associated with prognosis from GRNs would provide new insights into the progression of cancer and facilitate the translation of GRN-targeted therapies. Therefore, we designed a workflow to extract prognostic associated regulons by deconvoluting the patients’ cohorts with matched clinical data and bulk sequencing data. Here, we present and study a computational framework, termed as LPRI, to help prognostic stratification of patients. The prognostic model was tested and further validated in external datasets with the cross-platform cutoff. A mechanistic analysis was performed by utilizing the multi-omics data from TCGA. The association between LPRI and genomic alterations, the transcriptome landscape, and the immune microenvironment was implicated. In summary, this work provides a new insight into that LPRI could facilitate survival prediction and the development of personalized therapies for patients with LUAD.

## Materials and methods

### Patient cohorts in this study

Patient cohorts used in this study are summarized in Additional file 1: Table S1.

### Pre-processing of single-cell RNA sequencing data

The single-cell RNA sequencing dataset (GSE131907) was processed using Seurat (v4.0) in R v4.0.3 [9]. The annotation of cell types was provided by the authors. Firstly, the matrix of the filtered cells and genes was constructed as the input for Seurat. Cell filtering and gene selection were performed as mitochondrial genes (≤ 20%, unique molecular identifiers (UMIs), and gene count (from 100 to 150,000 and 200 to 10,000). We also excluded genes with min.cells < 0.1% cells. Then, we used the following parameters for the Seurat *ScaleData* function: do.scale = FALSE, do.center = TRUE, scale.max = 10. The mean gene expression variably expressed between 0.0125 and 3 and quantile-normalized variance greater than 0.5 were screened. Finally, through PCA visualization, the top 15 PCs were selected for the analysis, and the *RunUMAP* function was used for visualization.

### pySCENIC analysis

To identify the regulons in GSE131907, *SCENIC* algorithm was implemented by pySCENIC (v0.10.3) in python (3.6.11) [6]. The filtered single-cell expression matrix was taken as the input, and the co-expression module between TF and potential target genes was constructed and defined as a regulon. The module was inferred using GRNBoost2, and regulons were identified by RcisTarget. The regulon activity scores (RAS) for each cell was scored by AUCell. We next performed dimensions reduction by using UMAP with binarized AUCell score matrix of regulons as input.

### Inference of cell-type-specific regulons

To identify the cell-type-specific regulons in each type of cell in primary and metastatic LUAD, an entropy-based strategy developed by Suo et al. was adopted [10]. Briefly, this metric could measure the cell-type specificity score by Jensen–Shannon Divergence. The RAS was first normalized. The probability distribution which presented normalized RAS, was defined as:

$${P}^{R}=\left({P}_{1}^{R},\ldots ,{P}_{n}^{R}\right)$$

In this equation, n is the total number of cells, where:

$$\sum\limits_{i=1}^{n}{P}_{i}^{R}=1$$

Next, the cell-type-specific distribution which showed whether the cell was in a specific cell type or not was defined as:

$${P}^{C}=\left({P}_{1}^{C},\ldots ,{P}_{n}^{C}\right)$$

$$where\;\sum\limits_{i=1}^{n}{P}_{i}^{C}=1$$

$${P}_{i}^{C}=\left\{\begin{array}{ll}0 & {cell}_{i}\notin {{cell}\;{type}}^{C} \\ 1 & {{cell}}_{i}\in {{cell}\;{type}}^{C}\end{array}\right.$$

Then, we used the R package Philentropy (v0.5.0) to calculate Jensen-Shannon Divergence (JSD), widespread adopted for measuring the difference between two probability distributions, which was defined as:

$$JSD\left({P}^{R},{P}^{C}\right)=H\left(\frac{{P}^{R}+{P}^{C}}{2}\right)-\frac{H\left({P}^{R}\right)+H\left({P}^{C}\right)}{2}$$

In this equation, H means Shannon entropy of a probability distribution, defined as:

$$H\left(P\right)=-\sum {p}_{i}\;\text{log}\left({p}_{i}\right)$$

As a result, the regulon specificity scores (RSS) could be calculated by the following equation:

$$RSS=1-JSD\left({P}^{R},{P}^{C}\right)$$

$$RSS\in \left[{0,1}\right]$$

To a cell type C, the greater RSS value of regulon R was associated with the higher cell-type specificity. Meanwhile, we got binding motif logos from the JASPAR database (http://jaspar.genereg.net/).

### Construction of a gene signature from prognostic regulons

After identifying cell-type-specific regulons, the LUAD samples from TCGA (TCGA-LUAD) were considered as the training set for prognostic signature establishment. We filtered TFs and corresponding target genes, identified by the univariate cox regression analysis (P < 0.05). Next, the gene set variation analysis (GSVA), a non-parametric, unsupervised technique is used to estimate regulon enrichment scores, which was implemented by *GSVA* function in the R GSVA package (v1.38.2). The expression matrix and list of regulons were as input while keeping all other parameters at their default settings. As a result, we obtained TCGA-LUAD samples enrichment score for each regulon. The risk score was constructed by utilizing the regression coefficients derived from univariable Cox proportional hazards regression analysis to multiply the enrichment scores to help patients’ prognostic stratification, which we named as lung cancer prognostic regulon index (LPRI):

$$\text{LPRI}={\sum }_{i}coefficient \; \left(GSVA\;score\right) \times \left(GSVA\;score\right)$$

We next utilized it to construct survival analysis using maximally selected rank statistics to determine the cross-platform cutpoint, which divided the patients into the high-risk subgroup or the low-risk subgroup. As the LPRI model was established, the LUAD samples from the GEO database were used as the testing set. Univariate and multivariate Cox regression by ezcox (v1.0.0) in R was utilized to analyze these data sets [11]. Meanwhile, the prediction capability of the LPRI was further evaluated by plotting Kaplan–Meier curves and calculating the area under the ROC curve (AUC) by using R survivalROC (v1.0.3) and R timeROC (v0.4) packages.

### Clinical, transcriptome, epigenomic, genomic features analysis

The clinical traits were obtained from the TCGA-LUAD datasets, and we acquired TCGA transcriptome, epigenomic, and genomic features profiles from UCSCXenaShiny (v1.1.2) (https://openbiox.github.io/UCSCXenaShiny/reference/load_data.html), which were manually curated from previous TCGA publications [12]. Age was considered as an important factor [13]. According to the age categories in WHO and the mean age (65 years) in our cohort, we further stratify the patients into two groups (age < 60 and age ≥ 60). To interrogate subgroup-specific pathway activity, we performed pathway enrichment in each subgroup by using the Mann–Whitney–Wilcoxon Gene Set test (MWW-GST) [14]. The Enrichmap application of Cytoscape were used to visualize the pathway enrichment [15]. To integrate DNA methylation and RNA expression, we adapted the ELMER pipeline [16]. The CpG sites located at the promoter were firstly selected. We identified hypomethylated CpG sites (probes) between two subgroups with R *get.diff.meth* function. The nearby genes to CpG sites were identified with R *GetNearGenes* function. The gene-probe pairs were identified by R *get.pair* function. We performed the Wilcoxon rank-sum test (|log2FC| > 0 and FDR < 0.0005) to find out the differentially expressed miRNAs between two subgroups. To identify subgroup-specific functional miRNA targets, the R package miRNAtap was used and miRNA target genes whose expression were anti-correlated with miRNA expression in each subgroup were considered (Spearman’s correlation, ρ < 0 and p < 0.05). After prediction, we performed the Kyoto Encyclopedia of Genes and Genomes (KEGG) enrichment to distinguish two subgroups specific pathway functions through miRNA perspective by target genes. The relations between miRNAs, target genes and pathway functions were visualized by Cytoscape [17].

### Signature score estimation and immunotherapies analysis

The ESTIMATE algorithm could infer the overall infiltration levels of stroma and immune cells in tumor tissues using gene expression signatures. The expression data and clinical data from the IMvigor210 cohort were downloaded from http://research-pub.gene.com/IMvigor210CoreBiologies/ [18]. For characterization of the metabolism, immune microenvironment, and other gene signatures, we obtained gene signatures from IOBR package (https://github.com/IOBR/IOBR) and previous publications [19, 20].

### Prediction of chemotherapy drug sensitivity

Drug sensitivity data of human cancer cell lines were achieved from the Cancer Therapeutics Response Portal (CTRP v.2.0 and v.1.0) (https://portals.broadinstitute.org/ctrp) and PRISM Repurposing dataset (v.19Q4) (https://depmap.org/portal/prism/). Firstly, K-nearest neighbor (k-NN) imputation was applied to impute the missing AUC values by R impute (v1.68.0) package. Ridge regression was used in model training and predicting for drug sensitivity analysis, which was implemented by *calcPhenotype* function from the R pRRophetic (v0.5) package. Annotations for pathways of drugs are downloaded from Genomics of Drug Sensitivity in Cancer (GDSC, http://www.cancerrxgene.org/downloads) [21].

### Drug docking analysis

Docking analysis was performed based on the highly sensitive drugs predicted from the above analysis and the transcription factors gene list from LPRI. Firstly, we searched the Chemical-Gene Interaction in the CTD database (http://ctdbase.org/) to investigate whether the drugs could affect the expression of genes. Then, we downloaded the 3D structures of transcription factor proteins from the Uniprot database and the 3D structures of small molecule drugs from the PubChem database. After obtaining the spatial structure of the drug and target protein, we used AutoDock (v4.2.6) software for molecular simulation docking to determine the drug target based on the minimum binding energy, and used PyMOL (Version 2.4.0 Open-Source) for molecular docking visualization.

### Cell culture and antibodies

Human lung epithelial cells (BEAS-2B) and the lung cancer lines (A549, H358, PC9, 95 C, H520, SPCA-1 and H23) obtained from the Cancer Research Institute of Central South University were cultured respectively in DMEM (Gibco), DMEM/F-12 1:1 (Biohome) and RPMI 1640 (Gibco) medium supplemented with 10% fetal bovine serum (FBS), and all cells were maintained at 37 °C with 5% CO_2_. Additionally, these cell lines were subjected to detection of mycoplasma contamination and verified to be negative. Besides, all cell lines were passaged less than 10 times once revived from frozen stocks.

### Real-time quantitative PCR (RT-qPCR)

The total RNA of different lung cancer cell lines was isolated in accordance with the manufacturer’s instructions of RNAiso Plus (Takara clontech, Cat. 9109). Then cDNA of these cell lines was synthesized from RNA through reverse transcription reaction using PrimeScript™ RT reagent kit with gDNA Eraser(Takara clontech, RR047A). And finally, RT-qPCR assay was performed with 2× SYBR Green qPCR Master Mix (Bimake, B21203) and designed primers (Forward sequence: CTGCGCTCCAAGTACGAGGCG; Reverse sequence: TCGGTGGACTTGACGATGGTGA). The 2−ΔΔCT method was utilized to measure the relative mRNA expression of MAFK in different lung cancer cell lines.

### Immunoblotting (IB) assay

Immunoblotting assay was conducted as previously described. Briefly, the cells or tissue were harvested and lysed in IP buffer containing cocktail (protease inhibitor, bimake). The protein concentration was measured with Pierce™ BCA Protein Assay Kit (Thermo Scientific, Cat. 23225). Then, the protein samples were subjected to SDS-PAGE and immunoblotting with the indicated antibodies overnight at 4 °C. After washing three times with PBST buffer (10 min every time), the corresponding secondary antibody was added and incubated at 37 °C for another 1 h. Finally, the protein signal was detected using WesternBright Sirius Chemiluminescent Detection Kit (Advansta, Cat.k-12,043-D20) or WesternBright ECL kit (Advansta, Cat.k-12,045-D50).The following antibodies were obtained from commercial sources and used as primary antibodies: MAFK (GTX129240) was ordered from GeneTex, and β-actin (A5441) was gained from Sigma-Aldrich. Moreover, two kinds of secondary antibody, including anti-rabbit (7074) as well as anti-mouse (7076) IgG HRP-linked antibody, were obtained from CST.

### Statistics analysis

Continued variables between groups were compared by the Student’s t-test, one-way analysis of variance (ANOVA) test, or the Wilcoxon rank-sum test. Correlations between continuous variables were evaluated by Spearman or Pearson correlation analyses. For all statistical analyses, the P-value of 0.05 was taken as the significant threshold in all tests. All statistical analyses were performed using R software, version 4.1.1 (The R Foundation for Statistical Computing, http://www.rproject.org/) or Python.

## Result

### Construction of a scRNA-seq regulon atlas in LUAD

**Fig. 1:**
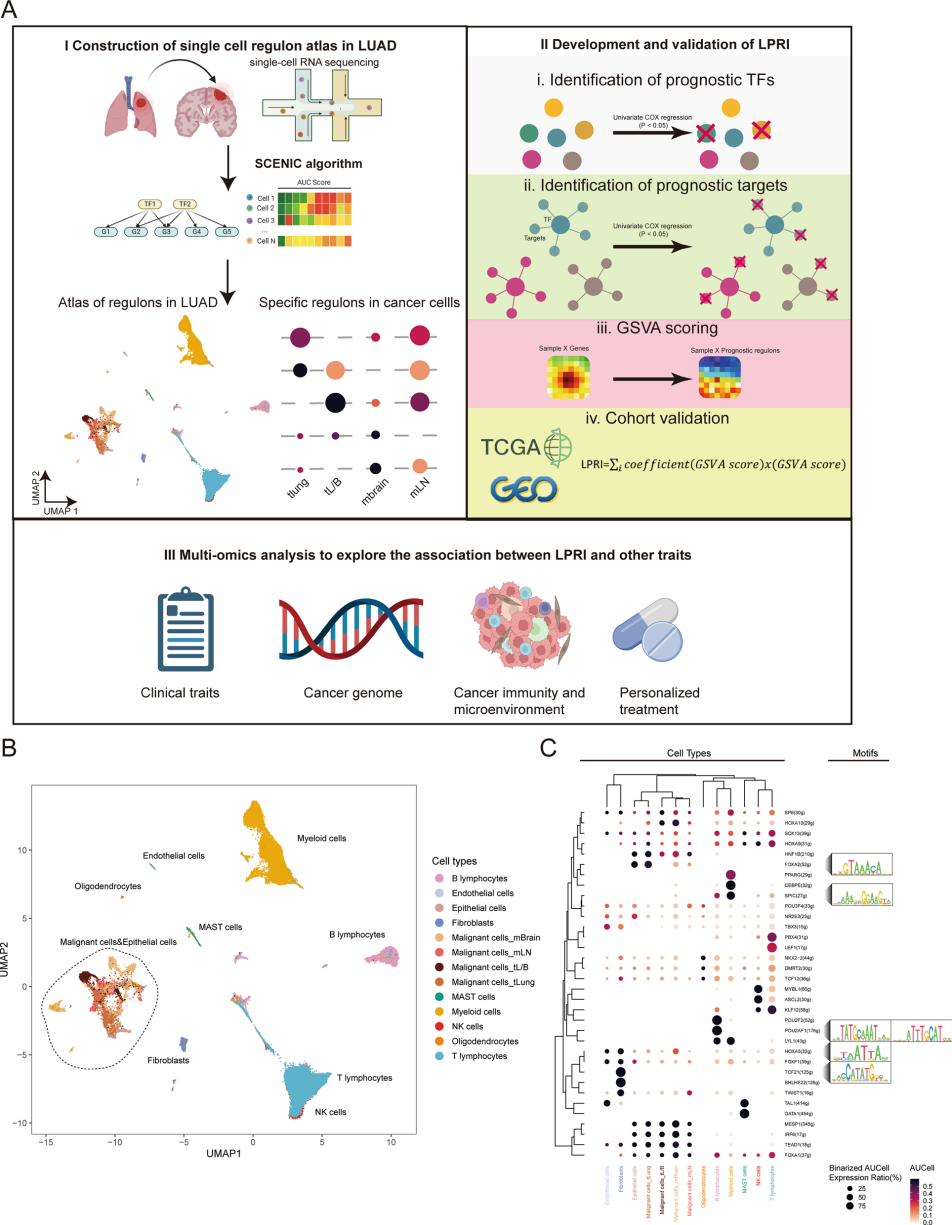
SCENIC analysis of human adenocarcinoma single cell dataset. **A** The workflow for this study. **B** Visualization of the SCENIC regulons from 208,506 cells from GSE131907 by using uniform manifold approximation and projection (UMAP). Each dot represents one cell and is colored according to cell types. **C** Bubble plot shows specific regulons across cell types. Bubble size is proportional to the percentage of cell types with the binarized AUCell expression, and color intensity is proportional to the scaled AUCell score within a cell type. The motifs of representative regulons are shown. *mBrain* brain metastases, *mLN* lymph node metastases, *tLung* and *tL/B*, primary tumors in lung or bronchus

The workflow of this study is outlined in Fig. 1A. To provide a comprehensive insight into the gene regulatory networks (GRNs) in LUAD, we first constructed a single-cell RNA-seq regulon atlas by leveraging the previously published dataset (GSE131907) (Fig. 1B). This dataset includes a total of 208,506 cells from 44 samples including the normal tissues and early to metastatic stage cancer. The major cell lineages include epithelial cells (malignant cells, non-malignant cells), stromal cells (fibroblasts and endothelial cells), immune cells (T, B, NK, and MAST cells), and oligodendrocytes in brain metastases. Next, we used the *SCENIC* algorithm to infer the regulon for each cell type, which infers co-expression modules between transcription factors (TFs) and candidate target genes using machine learning regression techniques, resulting in regulons. While some regulons are universally expressed across cell types, we found that many regulons are cell-type specific (Fig. 1C). For example, *POU2F2* regulon is shown to be associated with B cell proliferation and differentiation [22]. In agreement with the literature, *SPIC* regulon is highly enriched in myeloid lineage cells, which is required for the development of macrophages [23]. *TCF21* regulon is an essential regulator in fibroblast development [24]. Our results indicated that regulons could uncover the GRNs of each cell type in non-malignant cells. We also observed that malignant cells and epithelial cells are clustered together, indicating the epithelial origin of the most malignant cells (Fig. 1B). We then asked if the regulon could also reflect the intra-tumoral heterogeneity. It is intriguing that despite of some shared regulators, the variety of regulon expression indicates transcription reprogramming during metastasis. For example, the *FOXA2* regulon is expressed highly in the epithelial cells or malignant cells of the primary lung tumor, while its expression is decreased in tumors of the bronchus, which may be associated with epithelial-to-mesenchymal transition [25] (Fig. 1C). The expression of the *HOXA5* regulon is highly enriched in brain metastases, which may suggest its role in brain metastases. Taken together, our results indicate that our established single cell regulon atlas can faithfully uncover the heterogeneity of the GRNs in both malignant and non-malignant cells in LUAD (Fig. 1C).

### LPRI: a computational method to guide prognostic prediction for LUAD patients

**Fig. 2:**
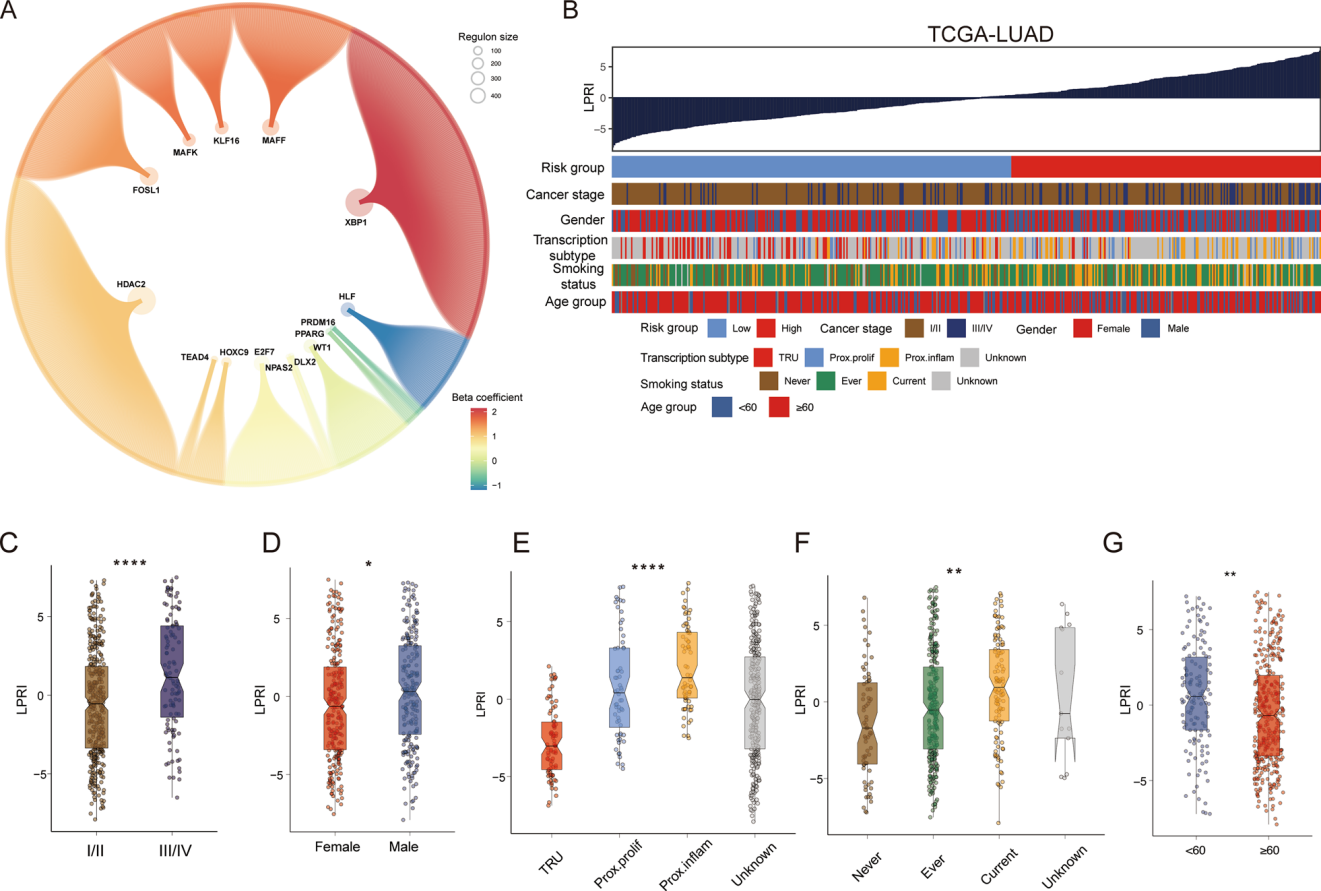
Development of LPRI and clinical features associated with LPRI. **A** Identification of prognostic regulons. Large bubbles represent the TFs. Small bubbles represent the targets. Bubble size is proportional to the number of target genes. Colors represent the beta coefficient for each regulon from the univariate COX regression analysis. **B** The clinical and molecular features associated with the LPRI in the TCGA-LUAD dataset. **C**–**G** Box plots of LPRI in individual samples, stratified by stage, gender, TCGA subtype, KPS, smoking status, and age. ‘ns’ means no statistical significance, ∗P < 0.05, ∗∗P < 0.01, ∗∗∗P < 0.001 and ∗∗∗∗P < 0.0001

**Fig. 3:**
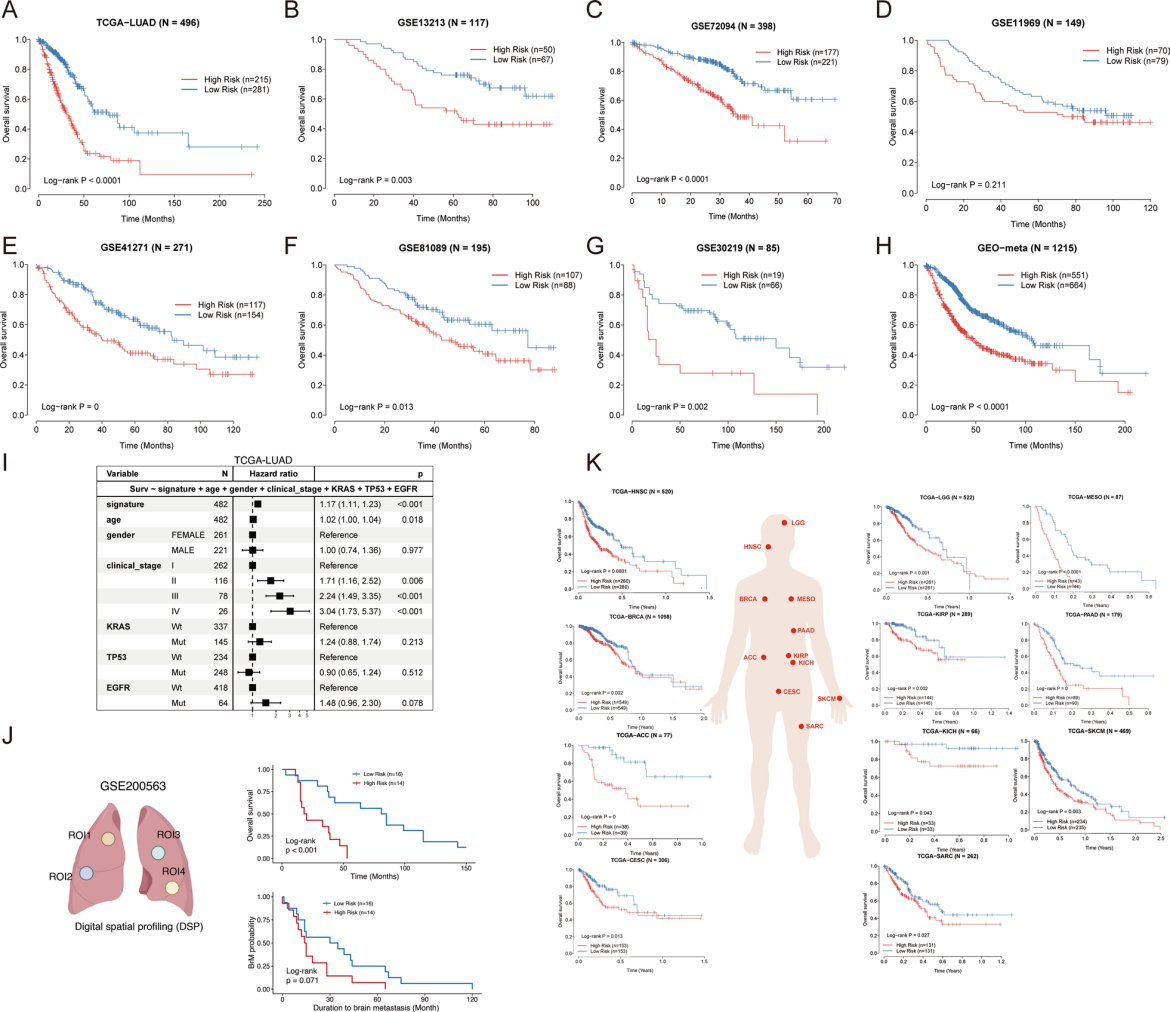
Prognostic value of LPRI. **A**–**H** Kaplan–Meier survival curves showing overall survival for patients grouped as high-and low-risk subgroups in the training set and validation set. **I** Multivariate Cox regression analysis for overall survival in the TCGA-LUAD dataset. **J** Kaplan–Meier survival curves in GSE200563. **K** LPRI stratifying survival probabilities in the TCGA-PANCAN dataset

The inter-tumor and intra-tumor heterogeneity would expectedly take place on the transcription level regarding cancer cells and the tumor microenvironment (TME). Here we focus on the intrinsic transcriptional heterogeneity of malignant cells. On the one hand, single-cell RNA sequencing has been limited to the cost and scarcity of samples. On the other hand, the bulk tumor expression data accompanied by clinical outcome metadata are abundant. Considering the advantages of scRNA-seq and bulk tumor sequencing data, we design the computational method to extract the core transcriptome program from the scRNA-seq data to guide patient stratification in large bulk tumor sequencing cohorts. We developed an algorithm to calculate an index for prognostic stratification from regulons, which we denote as the “Lung cancer Prognostic Regulon Index” (LPRI) (Fig. 1A). Briefly, we initially identified the top 20 regulons that are specific to each tissue site of malignant cells (lung, bronchus, lymph node metastases, and brain metastases) by adapting the method as previously published [10]. In total, 63 specific regulons were identified. Next, we performed a two-step feature selection to efficiently categorize clinical outcomes of LUAD patients via regulons. In the first step, we performed the univariate cox regression analysis on 63 transcriptional factor genes from the regulons. 15 transcriptional factor genes were identified as prognostic genes based on the criteria of P < 0.05. In the second step, we performed feature selection for target genes in the 15 regulons retained. Prognostic target genes in each regulon were identified by the univariate cox regression analysis (P < 0.05). Thus, we obtained a gene set that contains 15 prognostic-associated regulons (Fig. 2A). Finally, to calculate the enrichment of prognostic regulons, gene set variation analysis (GSVA) was performed to score each sample with these gene sets. We first calculated the LPRI using the TCGA-LUAD dataset as the training set. To determine a clinically useful cut-off value across patients, we used the maximally selected rank statistics method on the TCGA-LUAD training set. The optimal cut-off point to stratify patients was 0.33 (Additional file 2: Fig. S1A). We divided patients into two groups with this cut-off point. The patients with a higher LPRI had a lower survival probability. We named these patients as the high-risk subgroup. A systematic literature search was then performed to determine the inclusion of validation datasets in this study (Additional file 2: Fig. S1B). Subsequently, we used the cross-platform cut-off point 0.33 across validation datasets. High-risk subgroups showed consistently poorer clinical outcomes than low-risk subgroups (Fig. 3A–H). The prediction capability of the LPRI was further assessed by calculating the area under the ROC curves (AUCs). The AUCs of the LPRI for overall survival (OS) ranges from 0.7 to 0.96 for 1-year, 0.57–0.79 for 3-year, and 0.58–0.81 for 5-year in different datasets (Additional file 2: Fig. S2A–H). We also performed subgroup analysis based on the TNM stage system, because patients with early (clinical stage I and II) and advanced stage diseases (clinical stage III and IV) require different therapy strategies and hold different prognoses. We found that in both the early and advanced stages, LPRI could stratify patients (Additional file 2: Fig. S3A–D). We also observed that when patients are stratified by age, LPRI demonstrates good performance to predict patients’ survival (Additional file 2: Fig. S3E, F). In addition, we performed multivariate Cox regression analysis, which showed that LPRI was an independent prognostic factor after adjusting other clinical-pathologic features (Fig. 3I). Next, we also validated the survival associations of LPRI in an independent cohort (GSE200563) included 44 NSCLC patients in which different regions-of-interest (ROIs) were sequenced using the NanoString GeoMx DSP platform. We found that high-LPRI was associated with poor overall survival (P < 0.001) (Fig. 3J). Interestingly, we also found that high-LPRI patients have shorter duration to develop brain metastases (BrMs) (Fig. 3J). Pan-cancer analysis was performed to further validate the prognostic value of LPRI. We observed a significant association of LPRI and risk in multiple cancers including low-grade glioma (LGG) (Fig. 3K). Since an RNA-seq database from lung cancer brain metastasis with survival information is unavailable now, we found that high LPRI was associated with worse survival in LGG, which may have some shared features with BrMs.

### LPRI reclassify LUAD patients with distinct clinical features

The LPRI was ranked from low to high to explore the associations between LPRI and clinical traits in the TCGA-LUAD datasets (Fig. 2B). Patients had significantly elevated LPRI at the advanced stage (III/IV) (Fig. 2C). Regarding sex, male patients had higher LPRI than female patients (Fig. 2D). The previous study had proposed a transcriptome nomenclature: the terminal respiratory unit (TRU), the proximal-inflammatory (PI), and the proximal-proliferative (PP) [3]. The PP and PI subtypes had the highest LPRI (Fig. 2E). The PP and PI subtypes harbored *KRAS*, *TP53*, and *NF1* mutations were associated with a worse prognosis, which is consistent with our study [3]. LPRI was also elevated in smoking patients (Ever or current vs. never, all p < 0.05) (Fig. 2F). Additionally, we found that younger patients (age < 60) had elevated LPRI (Fig. 2G).

### LPRI contributes to distinct transcriptomic, epigenomic and miRNAs landscapes

**Fig. 4:**
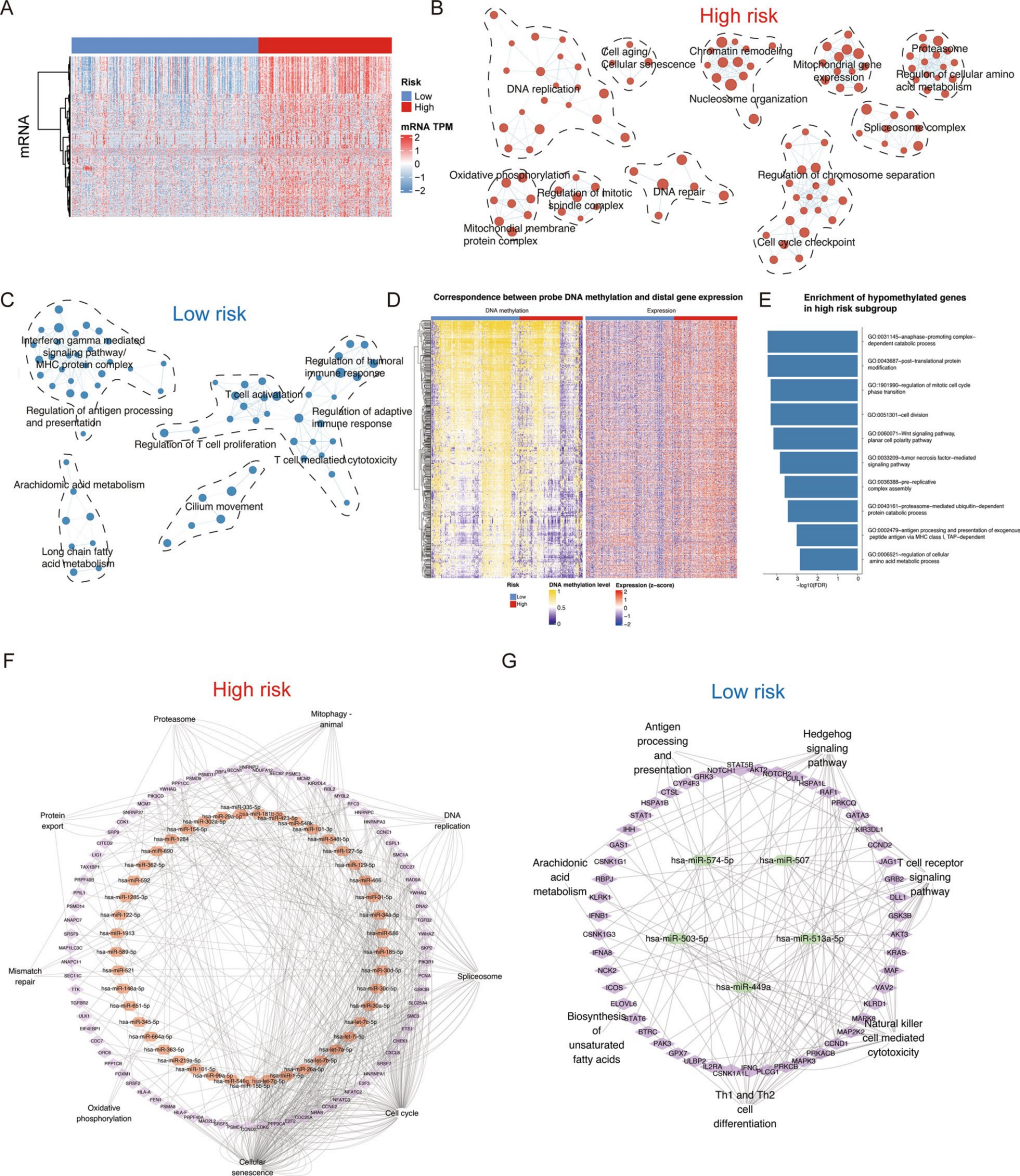
Characterization of transcriptome/epigenome features in LPRI subgroups. **A** The landscape of transcriptome profiles. **B**, **C** Enrichment map network of enriched GO categories in high-risk (**B**) or low-risk subgroup (Q value < 0.001, NES > 0.6) from two-sided MWW-GST analysis. **D** Identification of hypo-methylated probes and regulated genes. **E** GO enrichment of hypo-methylated genes in the high-risk subgroup. **F**, **G** Differentially expressed microRNA and the target genes in the high-risk (**F**) or low-risk subgroup (**G**)

Given the substantial difference of the two groups, we further explored the potential biological pathways linked with LPRI through the transcriptome (Fig. 4A). Analysis of the Gene Ontology enrichment map showed that pathways related to cell cycle, OXPHOS, DNA repair and the spliceosome complex were enriched in the high-risk subgroup while immune-related pathways and arachidonic acid metabolism/fatty acid metabolism pathways were enriched in the low-risk subgroup (Fig. 4B, C). As DNA methylation in the promotor usually anticorrelates with gene expression, we also investigated the methylomic differences in parallel with gene expression. We first identified Differentially Methylated CpGs (DMCs) occurring at promotor probes. Among them, 14,174 CpG sites exhibited hypomethylation in high-risk subgroup, while 631CpG sites were hypomethylated in low-risk subgroup. We next probed the nearby genes at these CpG sites and found significant probe-gene pairs. 2618 and 245 probe-gene pairs were identified for hypomethylation in the high-risk subgroup and low-risk subgroup, respectively. GO enrichment analysis of hypomethylated genes showed that pathways related to cell cycle regulation and post-translational protein modification were enriched in high-risk subgroup (FDR < 0.05) (Fig. 4E). However, we failed to identify statistically significant pathways enriched in low-risk subgroup. This suggests that the methylome and transcriptome may only have a synergistic effect in the high-risk subgroup. We next identified subgroup-specific miRNAs and functional miRNA targets. In the high-risk subgroup, we identified downregulated miRNAs which target genes associated with the cell cycle, spliceosome, proteasome, and cellular senescence related process (Fig. 4F). In contrast, the low-risk subgroup had low expression of several miRNAs that target genes associated with immune-related pathways, fatty acid metabolism and arachidonic acid metabolism (Fig. 4G). Overall, these results suggest that epigenetic and microRNA regulation coordinate with the transcriptome to mediate the different biological activities in different subgroups.

### Correlation of LPRI with the genome features

**Fig. 5:**
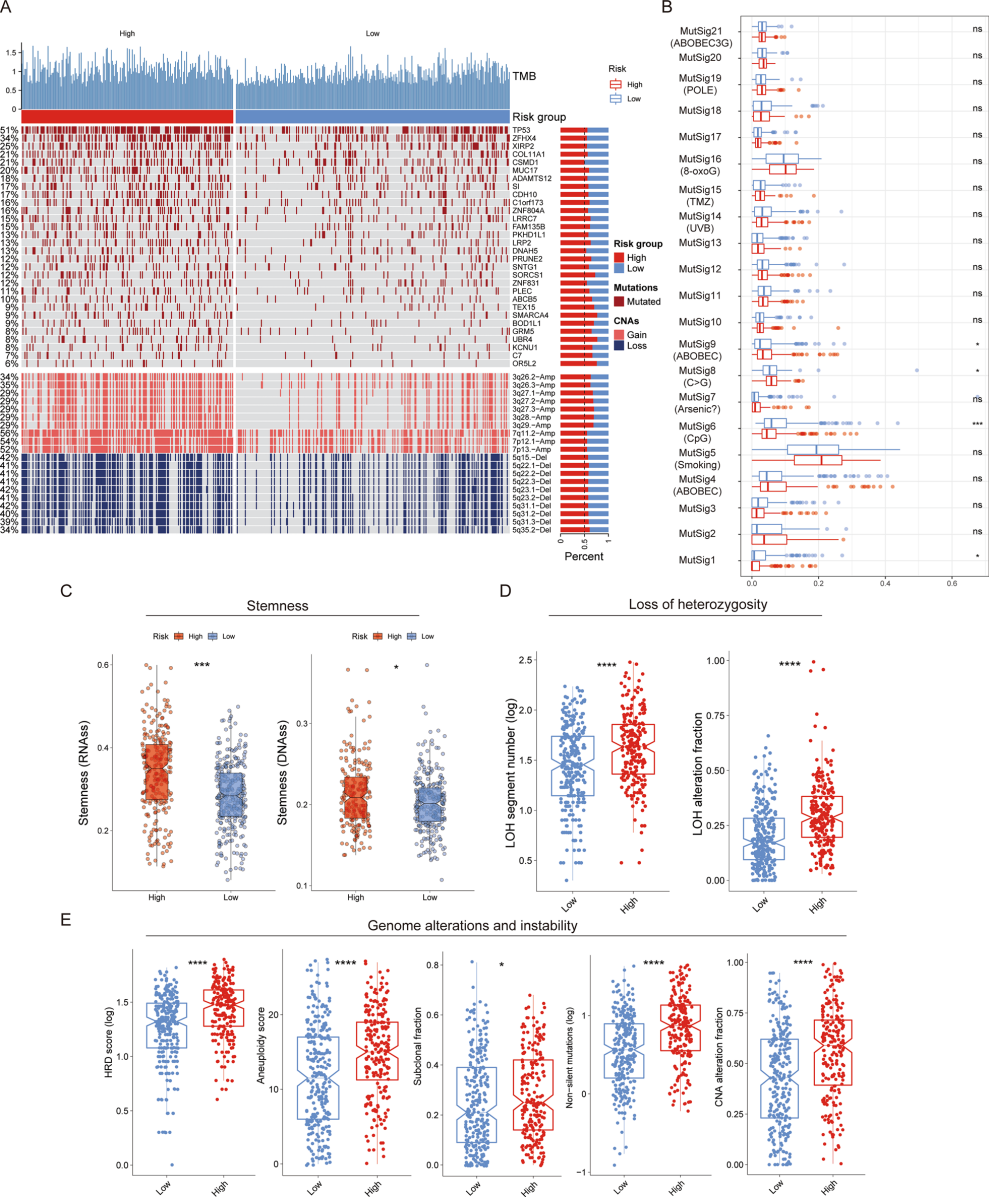
Characterization of genome alterations in LPRI subgroups. **A** Oncoprint shows the differentially altered gene mutations or CNV regions between high and low-risk subgroups. **B** Difference in mutational signatures between high and low-risk subgroups. **C**–**E** Comparisons of the stemness, loss of heterozygosity, genome alterations, and instability between low- and high-risk subgroups. ‘ns’ means no statistical significance, ∗P < 0.05, ∗∗P < 0.01, ∗∗∗P < 0.001 and ∗∗∗∗P < 0.0001

Somatic mutations and copy number variations (CNVs) are features of genome structures which impact gene expression. As we observed the dramatic difference in transcriptome between two subgroups, we further sought to explore the difference in genome features, which may partially explain the one in the transcriptome. Firstly, we found that the high-risk group had significantly higher non-silent tumor mutations, which indicates higher tumor mutation burdens (TMBs). Of the most frequently altered genes, the high-risk group had the most frequent mutations in *TP53*, *TTN*, *CSMD3*, *MUC16* genes, while the low-risk group had the most frequent mutations in *TP53*, *TTN*, *MUC16*, *RYR2* genes (Fig. 5A). Of differentially altered genes, *TP53* is a known tumor suppressor. *ZFHX4* is a transcription factor and has been shown to be associated with the maintenance of tumor-initiating cells in glioblastoma [26, 27]. *XIBP2* encodes an actin-binding protein and its mutations in *XIBP2* were observed frequently in human lung adenocarcinoma [3]. However, the mechanism behind how mutations in this gene arise in cancers remains unclear [28]. Therefore, further investigation of the role of this gene and other differentially mutated genes in human cancers is still warranted. Somatic mutations in the cancer genome could be caused by both exogenous and endogenous factors. Mutational signatures had been used to categorize the mutational patterns. Thus, potential exogenous and endogenous factors could be linked with the mutational patterns via mutational signatures. Mutational signature analysis revealed that MutSig5 (associated with smoking) had the highest score among all signatures. We found that the MutSig6 score was significantly different between the high-risk and low-risk groups, which was associated with CpG islands, indicating the potential epigenetic differences. MutSig9 was associated with ABOBEC mutagenesis, the difference of which between the high and low-risk subgroups may suggest various immune-associated activities (Fig. 5B). In addition to the somatic mutations, the high-risk subgroup had significantly higher copy number loss or gain, especially the 3q26, 3p27, 3q28, 3p29, 7p11, 7p12, 7p13 amplifications and 5q15, 5q22, 5q23, 5q31, 5q35 deletions. Chromosome 3q amplification was associated with lung cancer progression [29]. The higher frequency of 3q amplifications in the high-risk group may explain the deteriorated prognosis in this group. Moreover, we also found higher loss of heterozygosity, homologous recombination deficiency (HRD) score, aneuploidy score, sub-clonal fraction, and CNA alteration fraction in the high-risk subgroup, indicating higher genome instability (Fig. 5C–E). The substantially higher stemness indices also revealed the tumor aggressive phenotype in the high-risk subgroup, which also indicates higher tumor metastases propensity [30]. We further explored the difference of genomic features stratified by patient age. We observed substantial difference in mutation frequency of several genes such as *TP53* (64% in patients < 60 vs. 47% in patients ≥ 60) (Additional file 2: Fig. S4A, B). We also observed that loss of heterozygosity, homologous recombination deficiency (HRD) score and CNA alteration fraction are higher in the high-risk subgroup in both age groups. Moreover, we found that aneuploidy score and sub-clonal fraction are higher in the high-risk subgroup only in patients ≥ 60. These analyses indicate age could be considered as a factor which might contribute to the genome instability (Additional file 2: Fig. S4C–M).

### Correlation of LPRI with the TME and immunotherapies

**Fig. 6:**
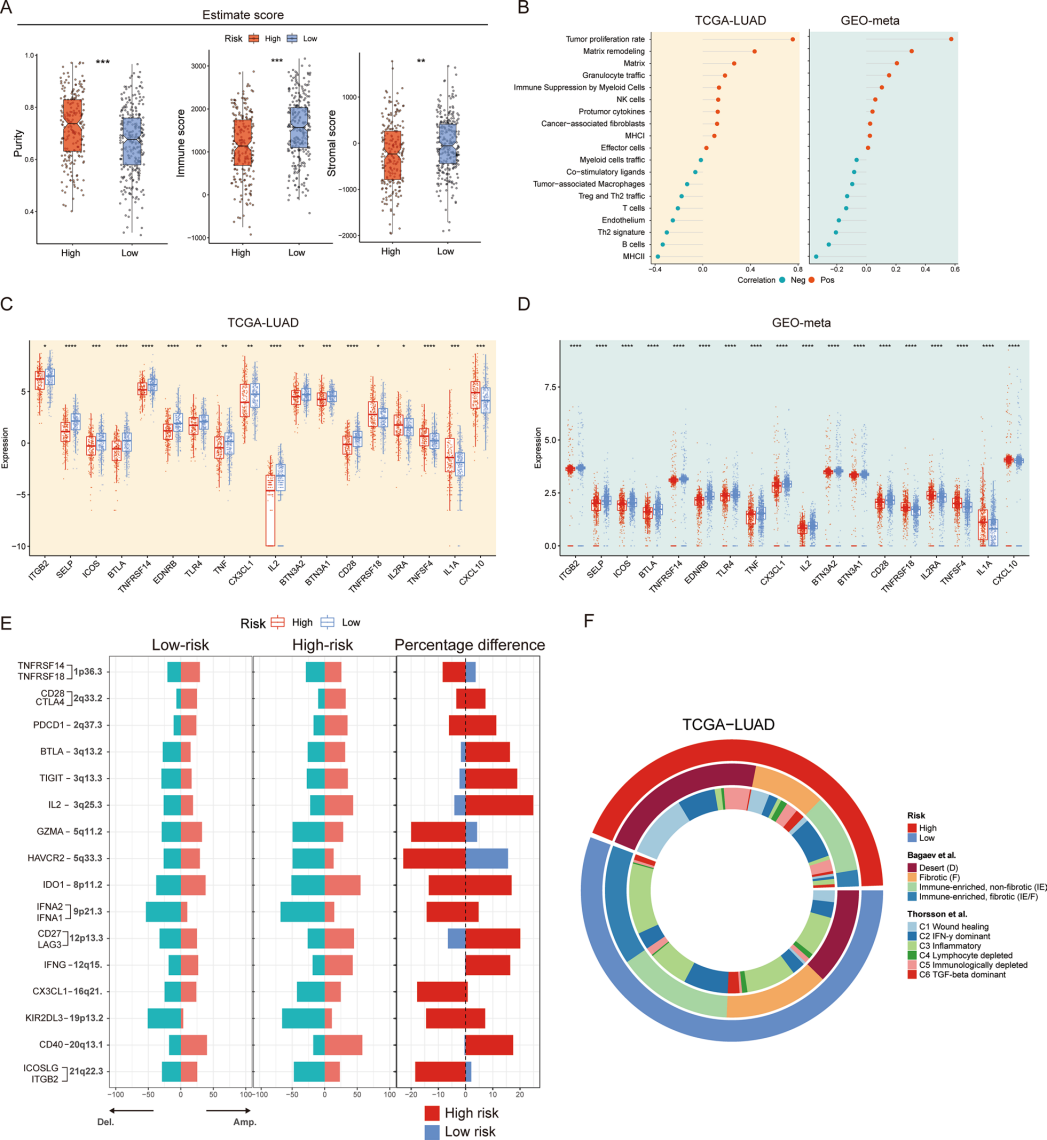
Characterization of immunogenomic features in LPRI subgroups. **A** Comparisons of the Purity, ImmuneScores, and StromalScores from the ESTIMATE software between low- and high-risk subgroups. **B** Pearson’s correlation between LPRI and TME signatures in the TCGA-LUAD and GEO-meta dataset. **C**, **D** Differences in immune genes between and GEO-meta dataset. **E** Altered CNV between low- and high-risk subgroups in the TCGA-LUAD in immunogenomics. **F** Comparison of risk subgroups in the current study with immune subtypes from previous studies. ‘ns’ means no statistical significance, ∗P < 0.05, ∗∗P < 0.01, ∗∗∗P < 0.001 and ∗∗∗∗P < 0.0001

The significant correlations between LPRI and immune-related pathway alterations prompt us to hypothesize that different risk subgroups may link to altered tumor microenvironments (TME). The association between ESTIMATE scores of tumor immune infiltrates as well as immune checkpoint expression between subgroups were further explored. ESTIMATE scores could be used to infer the fraction of stromal and immune cells in solid tumors. ESTIMATE purity was significantly elevated in the high-risk subgroup, while the ImmuneScores and StromalScores were higher in the low-risk subgroup (Fig. 6A). These results implicate higher tumor signaling in the high-risk subgroup while higher immune/stromal signaling in the low-risk subgroup. Gene signatures that reflect the TME, tumor-TME interaction, and tumor behavior were further evaluated. We found that the high-risk subgroup is associated with tumor proliferation, and matrix remodeling process, whereas the low-risk subgroup highly expresses T cells, endothelium, and Th2 signature (Fig. 6B). Next, we checked the expression of immune checkpoint genes. The differential expression of immune checkpoint genes indicates the dynamics of TME (Fig. 6C, D). Further, we hypothesize that the different immunogenomics would contribute to the TME difference. Genomic alterations may impact TME activity. Subsequently, links between CNVs and subgroups were studied. We mapped the immunoregulatory genes onto the altered CNV regions. We found that CNVs may contribute to the alterations in the transcriptome for some immunoregulatory genes (Fig. 6E). For example, *TNFRSF14*, *ITGB2*, and *CX3CL1* (locate at 1p36.3, 21q22.3, and 16q21, respectively) were highly expressed in the low-risk subgroup while deletions in these regions were observed more frequently in the high-risk subgroup. *TNFRSF14* encodes a member of the TNF (tumor necrosis factor) receptor superfamily, which can function as an inflammatory activator. *ITGB2* encodes an integrin beta chain, which can be involved in cell-cell interactions. *CX3CL1* encodes proteins in the CX3C subgroup of chemokines, which is a regulator in chemokine activity. Alterations in other immunoregulatory genes in the transcriptome level may not directly associate with CNVs, suggesting possibly more complex regulatory mechanisms.

Finally, we checked the concordance of our subtype and previously reported subtypes (Fig. 6F). The high-risk subgroup is associated with the immune-desert subtype, while the low-risk subgroup is associated with the immune-riched, fibrotic subtype [19]. Additionally, the high-risk subgroup is associated with the wound-healing subtype while the low-risk subgroup is associated with the inflammatory subtype [31].

### The potential therapeutic value of stratification of LUAD patients with LPRI

**Fig. 7:**
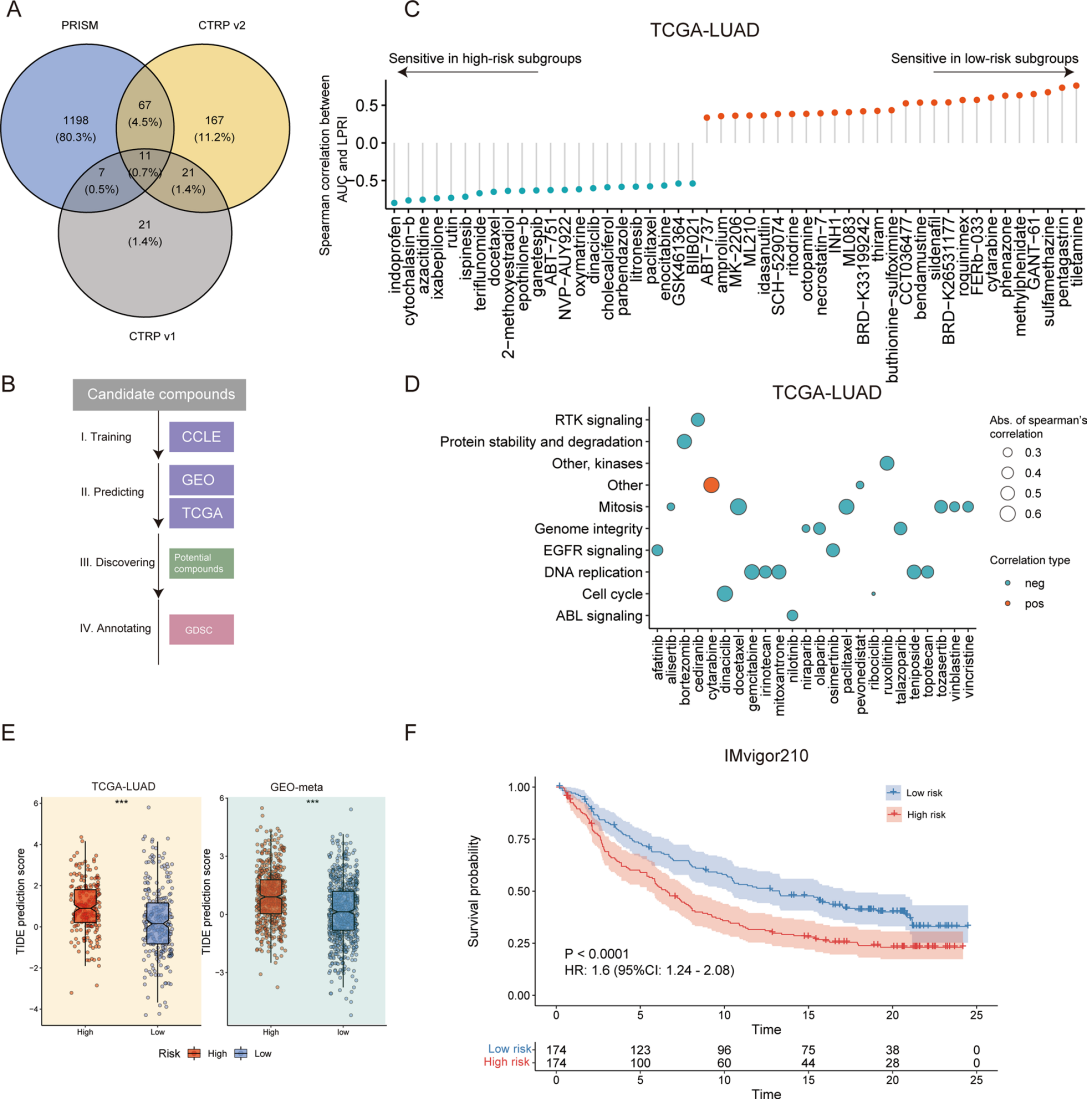
LPRI predict drug response in the chemotherapy and immunotherapy. **A** Veen plot shows the number of potential drug candidates from three databases, PRISM, CTRP v1, and CTRP v2. **B** Strategy for discovering potential sensitive drugs associated with LPRI. **C** Spearman’s correlation between LPRI and predicted AUCs of drugs. **D** Pathway annotation of potential sensitive drugs. **E** TIDE prediction scores between low- and high-risk subgroups. **F** Kaplan–Meier curves of overall survival in the high and -low (blue) subgroups based on LPRI after the PD-L1 blockade immunotherapy in the IMvigor210 cohort. ‘ns’ means no statistical significance, ∗P < 0.05, ∗∗P < 0.01, ∗∗∗P < 0.001 and ∗∗∗∗P < 0.0001

Next, we sought to investigate the potential treatment value for patients stratified with LPRI. We first retrieved drug response data from three databases: PRISM, CTRP v1, and CTRP v2. A total of 1492 compounds were collected (Fig. 7A). We then used ridge regression to train a model with CCLE datasets which contain drug response data in cancer cell lines. We next utilized the pre-trained model to predict drug sensitivity in the TCGA-LUAD and GEO-meta datasets. A lower AUC value is associated with higher drug sensitivity (Fig. 7B). We identified 477 correlated pairs and 802 correlated pairs between LRPI and predicted AUC values in the TCGA-LUAD and GEO-meta datasets, respectively. Compounds with the most different AUC values are shown in Fig. 7C and Fig. S4A. We found that drugs targeting mitosis (docetaxel, paclitaxel, et al.) or DNA replication signaling (gemcitabine, mitoxantrone, et al.) were associated with high sensitivity in LRPI-high patients (Fig. 7C, D and Additional file 2: Fig. S5A, B).

On top of chemotherapy, therapies targeting immune pathways have revolutionized the treatment of cancers. We sought to investigate whether LPRI could be used to predict efficacy in immunotherapies. We first obtained the TIDE prediction score for two datasets. TIDE prediction score correlates with T cell dysfunction and exclusion. A higher TIDE prediction score is associated with a worse response from immunotherapies [32]. We found that the high-risk subgroup had a lower TIDE prediction score indicating less sensitivity to the immunotherapy (Fig. 7E). Furthermore, we surveyed the publicly available databases including GEO, and the literature. However, the pre-treatment whole transcriptomics files together with therapy response or survival of lung adenocarcinoma patients are unavailable due to limited sample size or limited access to data. Alternatively, we further validated this in the IMvigor210 dataset, which comprises 378 patients receiving atezolizumab (anti-PD-L1) in metastatic urothelial cancer. We found that LPRI could stratify patients by survival probability from the IMvigor210 cohort (Fig. 7F). The patients with progression diseases (PD) showed higher LPRI than patients with other types of responses (Additional file 2: Fig. S5C). We also observed that LPRI of the “immune inflamed” type was higher than the “immune excluded” type (Additional file 2: Fig. S5D). In summary, these results indicate that LPRI can be used as a biomarker for treatment stratification and to build appropriate treatment options.

### LPRI-aided identification of potential cancer biomarkers in LUAD

**Fig. 8:**
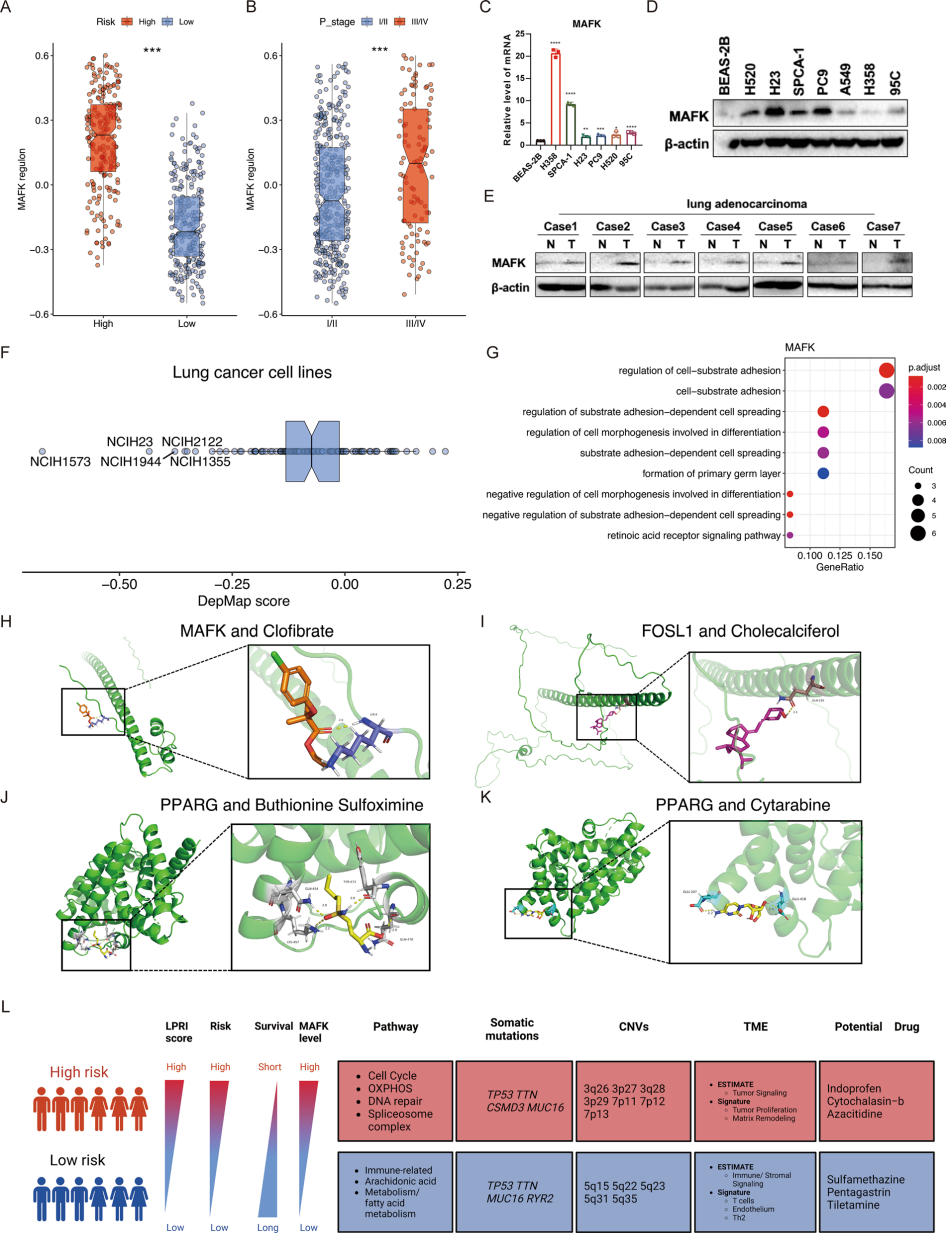
MAFK serves as a biomarker in LUAD. GSVA score of MAFK regulon between low- and high-risk subgroups (**A**) or clinical stages (**B**) in the TCGA-LUAD dataset. **C** RT-qPCR detection of MAFK expression in normal BEAS-2B cell line and lung cancer cell lines. **D** Western blot detection of MAFK expression in normal NP69 cell line and lung cancer cell lines. **E** Western blot detection of MAFK expression in patients’ cancerous tissue and adjacent normal tissue. **F** DepMap score of MAFK in different lung cancer cell lines. **G** GO enrichment analysis of genes in MAFK regulon. **H**–**K** Visualization of the docking results of proteins encoded by prognostic genes with small molecular compounds. **L** Summary of this study. ‘ns’ means no statistical significance, ∗P < 0.05, ∗∗P < 0.01, ∗∗∗P < 0.001 and ∗∗∗∗P < 0.0001

In this study, we identified 15 prognostic regulons, which could serve as candidates for further exploration of their function in tumorigenesis (Fig. 2A). As a proof of concept, we focused on *MAFK*. Our findings showed that the MAFK regulon was highly expressed in the high-risk subgroup and advanced stage (III and IV) in LUAD (Fig. 8A, B). To further explore the significance of *MAFK* in LUAD, RT-qPCR and Immunoblotting assays were performed to detected the expression of *MAFK* both at the mRNA and protein level in human normal lung epithelial cells (BEAS-2B) and various lung cancer cell lines. The results demonstrated that MAFK was highly expressed in cancer cell lines at the RNA and protein level, although the expression levels varied across cell lines (Fig. 8C, D). MAFK was highly expressed in paired cancerous tissue compared to adjacent normal tissue in human patients’ samples (Fig. 8E). MAFK could also serve as a cancer essential gene in several lung cancer cell lines (Fig. 8F) from the Depmap dataset [33]. GO enrichment analysis revealed that MAFK may regulate cell-substrate adhesion (Fig. 8G). Further functional validation of the mechanism of MAFK in tumorigenesis is warranted.

After conducting the drug sensitivity analysis and obtaining a list of potential drugs, we wanted to determine whether and how these drugs could affect the expression of genes in LPRI. Using a simulated approach, we pinpointed drugs that specifically target prognostic transcription factors. For instance, we found that Clofibrate was shown to effectively decrease the mRNA expression level of MAFK (docking energy of − 1.98 kcal/mol). Clofibrate is a medication used to lower lipid levels in the bloodstream, specifically targeting high levels of cholesterol and triacylglyceride (Fig. 8H). FOSL1 was also a predictor for worse prognosis. We also found that Cholecalciferol, a type of vitamin D, could result in decreased expression of FOSL1 mRNA, also had ability to bind to FOSL1, with binding energy of − 3.67 (kcal/mol) (Fig. 8I). Our study has indicated that high expression of PPARG is a good prognostic indicator. PPARG has been shown to inhibit cancer cell proliferation and induce apoptosis [34]. We found that the chemotherapy adjuvant drug Buthionine Sulfoximine and the commonly used chemotherapy drug Cytarabine can also promote the expression of PPARG mRNA (Fig. 8H, I). Docking simulation analysis for PPARG protein and these drugs has shown that the binding energy of Buthionine Sulfoximine with PPARG is − 3.6 kcal/mol, and the binding energy of Cytarabine with PPARG is − 2.73 kcal/mol. Our analysis found that these drugs have the potential to specifically bind to the PPARG protein.

## Discussion

Dysregulation of regulatory programs is a significant factor in tumor development. Single-cell omics studies increasingly demonstrate that intratumor heterogeneity is a crucial marker of tumor development. We utilized scRNA-seq data from GSE131907 to construct an atlas of a single-cell regulatory network for LUAD via the SCENIC algorithm, comprising a total of 208,506 cells. We identified cell-specific regulatory programs and hypothesized their association with tumor development and clinical relevance. As such, we developed an algorithm to extract prognostic-associated regulons from bulk gene expression profiles and single-cell sequencing datasets. To aid patient stratification, we created an index, LPRI, which we validated in both training and validation datasets. We then conducted a comprehensive investigation of the landscapes of the tumor transcriptome, tumor genome, and TME of stratified patients in the TCGA-LUAD dataset. We observed significant alterations in genomics, biological pathways, and TME among different groups, indicating treatment vulnerability for various subgroups (Fig. 8L).

In this study, we proposed an index, LPRI, consisting of 15 prognostic-associated regulons. To gain insight into the biological significance of this gene signature, we discuss the core transcription factor genes included in this gene signature. Several candidates have been reported to play a role in tumorigenesis in lung cancer or other cancer types. For instance, XBP1, which regulates the unfolded protein response (UPR) during endoplasmic reticulum (ER) stress, promotes NSCLC tumorigenesis, invasion, and metastasis by regulating the IGFBP3/MMP-9 axis [35]. Additionally, IRE1α-XBP1 signaling is essential for NSCLC progression, and ablation of IRE1α-XBP1 signaling extends survival in vivo by eliciting adaptive anti-cancer immunity [36]. The CREB3 family of ER-localized, bZIP transcription factors has been implicated in the ER and Golgi stress responses as regulators of the cell secretory capacity and cell-specific cargos [37]. SEC61G participates in endoplasmic reticulum stress by interacting with CREB3 to promote the malignant progression of lung adenocarcinoma [38]. Krüppel-like factors (KLFs) are DNA-binding transcriptional regulators involved in various cellular processes, including proliferation, migration, inflammation, and angiogenesis [39]. KLF16 overexpression promoted lung cancer cell growth and invasion [40]. The Maf proteins (Mafs) belong to the activator protein-1 (AP-1) superfamily and are basic leucine zipper transcription factors. MafG, MafF and MafK belongs to small Mafs (approximately 150–160 amino acids) [41]. MafG accelerates cell proliferation and inhibits cell apoptosis in lung adenocarcinoma [42], while MafF promotes tumor invasion through heterodimerizing with Bach1 and activating the IL11/STAT3 pathway in breast cancer [43]. MafK Induces EMT and promotes tumor invasion in vivo in breast cancer [44]. Our study reveals that MafK is essential for LUAD tumorigenesis and is highly expressed in cancer tissue/cell lines. Bioinformatics analysis indicates that MafK promotes tumor invasion via the cell-substrate adhesion pathway.

Moreover, we divided all samples into low- and high-risk groups based on the LPRI. We observed that the low-risk group was primarily enriched in immune processes and immune-related pathways, suggesting that the risk score could serve as a potential predictive indicator for patients undergoing immunotherapy. To investigate this further, we evaluated immune infiltration gene signature, immune checkpoints, and associated inflammatory genes from different perspectives. Our findings indicated that low-risk groups are associated with T cell gene signature and have high expression levels of immunoregulatory genes such as TNFRSF14, ITGB2, and CX3CL1. Moreover, we observed that the low-risk groups are associated with the immune-rich, fibrotic subtype or inflammatory subtype, indicating that these patients have higher immune activity.

Lastly, we identified potential druggable targets and corresponding compounds for LUAD patients using the PRISM and CTRP databases based on the developed prognostic models. We predict that drugs will have varying sensitivity in different subgroups of patients. Using molecular docking analysis, we discovered that some drugs can target prognostic transcription factors specifically. Although these drugs have not yet been adopted in the clinic, they hold great promise as future antitumor drugs.

While our work initially focuses on the stratification of lung adenocarcinoma patients, the algorithm presented here is applicable to other cancer types and non-cancerous diseases. We acknowledge the limitations of this study, namely that the prognostic efficacy of LPRI was tested in retrospective cohorts, and prospective studies are required to validate its clinical utility. Further investigation into the functional mechanisms of regulons in cancers is also warranted.

## Conclusion

To summarize, this study established a single-cell regulon atlas in human lung adenocarcinoma (LUAD) and developed a workflow called LPRI based on the integration of single-cell regulon and clinical bulk sequencing cohort. This led to a significant advancement in the prognostic classification of LUAD. The integrated multi-omic analysis revealed that the two subgroups based on LPRI had different survival outcomes, tumor genomics, tumor microenvironment, and different responses to chemotherapy and immunotherapy. These findings have important clinical implications as they provide a more comprehensive understanding of the heterogeneity of LUAD and the potential for personalized treatment strategies. Further research is required to validate these findings and to explore additional markers that could enhance the accuracy of LUAD prognostic classification.

## Supplementary Information

**Additional file 1: Table S1.** Basic information of included datasets. **Table S2.** Identification of prognostic regulons. **Table S3.** LPRI of all included datasets.

**Additional file 2: Figure S1.** Workflow for identification of optimal cut-off for LPRI and data set selection. **Figure S2.** The time-dependent ROC curves for overall survival. **Figure S3.** Kaplan-Meier survival curves or multivariate Cox regression analysis stratified by stage. **Figure S4.** Characterization of genome alterations in LPRI subgroups stratified by age. **Figure S5.** LPRI predict drug response in the chemotherapy and immunotherapy in the GEO-meta dataset.

## Data Availability

The datasets used and/or analyzed during the current study are available from the corresponding author on reasonable request. To facilitate using of our developed workflow by the community, an R package has been developed (https://github.com/Richard-Li-lab-team/LPRI).

## Abbreviations

ANOVA: One-way analysis of variance
CNV: Copy number variations
EMT: Epithelial–mesenchymal transition
GRNs: Gene regulatory networks
GSVA: Gene set variation analysis
k-NN: k-Nearest neighbor
LUAD: Lung adenocarcinoma
LPRI: Lung cancer prognostic regulon index
MAF: Musculoaponeurotic fibrosarcoma
NSCLC: Non-small cell lung cancer
PP: Proximal-proliferative
PI: Proximal-inflammatory
TRU: Terminal respiratory unit
TF: Transcription factor
TCGA: The Cancer Genome Atlas
TME: Tumor microenvironment
TMB: Tumor mutation burden
UMI: Unique molecular identifiers
UPR: Unfolded protein response
