# Supplementary material for: Integration of single-cell regulon atlas and multi-omics data for prognostic stratification and personalized treatment prediction in human lung adenocarcinoma
Source: J Transl Med. 2023 Jul 25;21:499. doi: 10.1186/s12967-023-04331-z (PMC10369768; doi:10.1186/s12967-023-04331-z)
Supplement: Supplementary file 2 — Additional file 2: Figure S1. Workflow for identification of optimal cut-off for LPRI and data set selection. Figure S2. The time-dependent ROC curves for overall survival. Figure S3. Kaplan-Meier survival curves or multivariate Cox regression analysis stratified by stage. Figure S4. Characterization of genome alterations in LPRI subgroups stratified by age. Figure S5. LPRI predict drug response in the chemotherapy and immunotherapy in the GEO-meta dataset. [file 12967_2023_4331_MOESM2_ESM.docx]

**Integration of single-cell regulon atlas and multi-omics data for prognostic stratification and personalized treatment prediction in human lung adenocarcinoma**

Yi Xiong^1,2,3,*^, Yihao Zhang^1,2,3,*^, Na Liu^1,2,4^, Yueshuo Li^1,2,4^, Hongwei Liu^1,2^, Qi Yang^1,2^, Yu Chen^3^, Zhizhi Xia^5^, Xin Chen^6,#^, Siyi Wanggou^1,2,#^, Xuejun Li^1,2,#^

1 Department of Neurosurgery, Xiangya Hospital, Central South University, Changsha, Hunan, 410008, China

2 Hunan International Scientific and Technological Cooperation Base of Brain Tumor Research, Xiangya Hospital, Central South University, Changsha, Hunan, 410008, China

3 Xiangya School of Medicine, Central South University, 410013 Changsha, China

4 Postdoctoral Research Workstation, Xiangya Hospital, Central South University, Hunan, 410078, China

5 Department of Pharmacology & Toxicology, University of Toronto, Toronto, Ontario M5S 1A8, Canada

6 Songjiang Research Institute, Songjiang Hospital, Shanghai Jiao Tong University School of Medicine

* These authors contributed equally to this work.

# Author for correspondence:

Xuejun Li, [lxjneuro@csu.edu.cn](mailto:lxjneuro@csu.edu.cn)

Siyi Wanggou, [zokygwong@gmail.com](mailto:zokygwong@gmail.com)

Xin Chen, xinchen@shsmu.edu.cn

**Supplementary Figures**

Figure S1


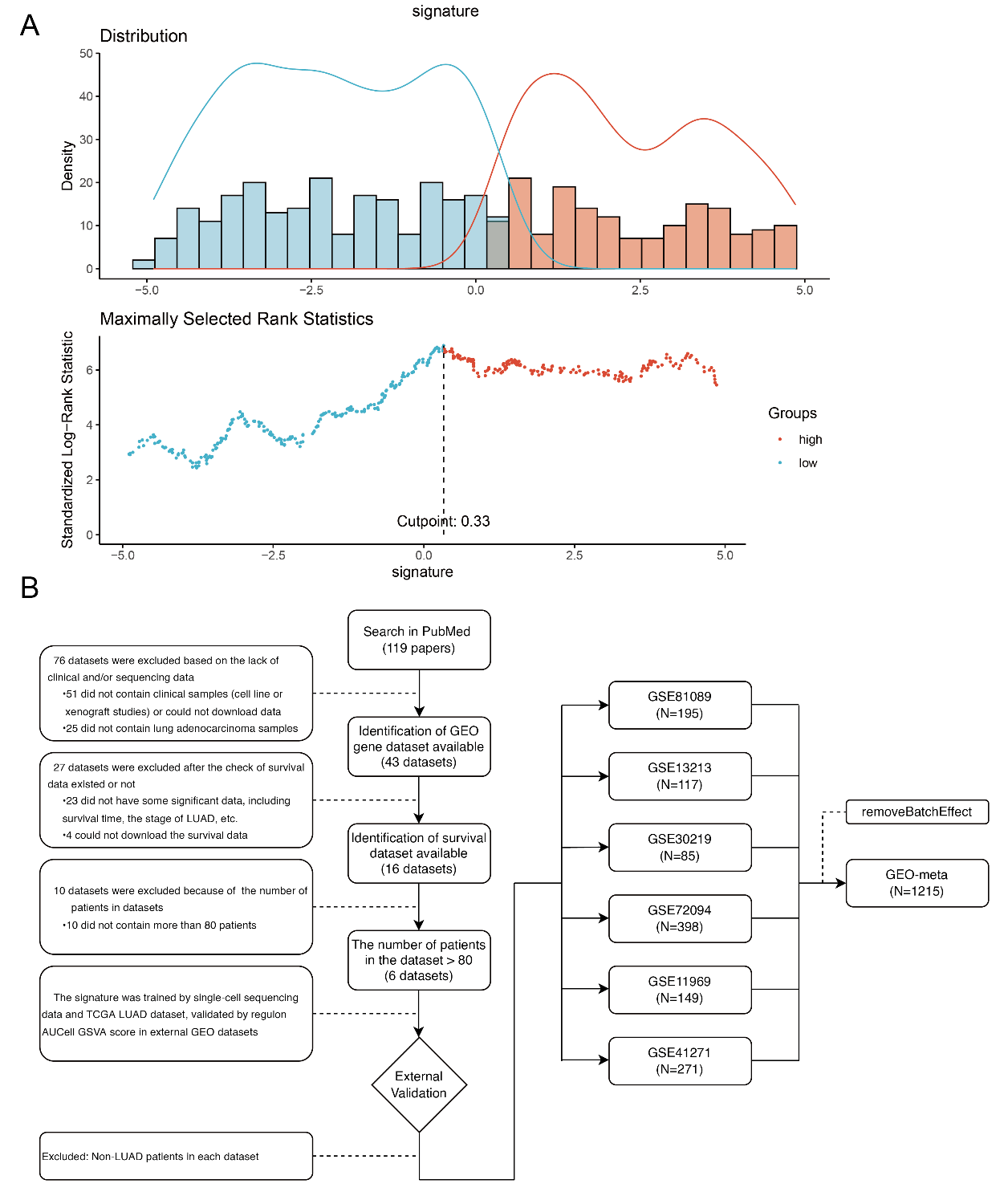


**Workflow for identification of optimal cut-off for LPRI and data set selection.** The optimal cut-off value to divide LPRI as low and high subgroups in the TCGA-LUAD dataset (a). Flow diagram for data set selection in this study (b).

Figure S2


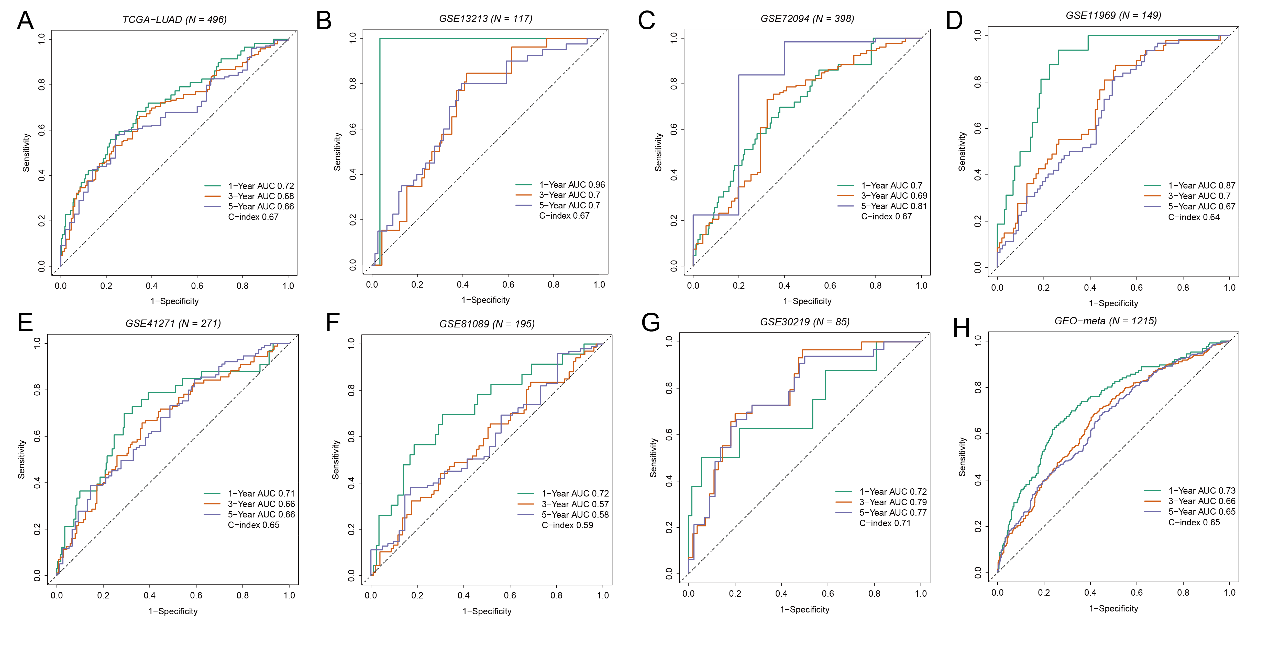


**The time-dependent ROC curves for overall survival.** The time-dependent ROC curves for 1-, 3-, and 5-year overall survival in the training set and validation sets (a-h)

Figure S3


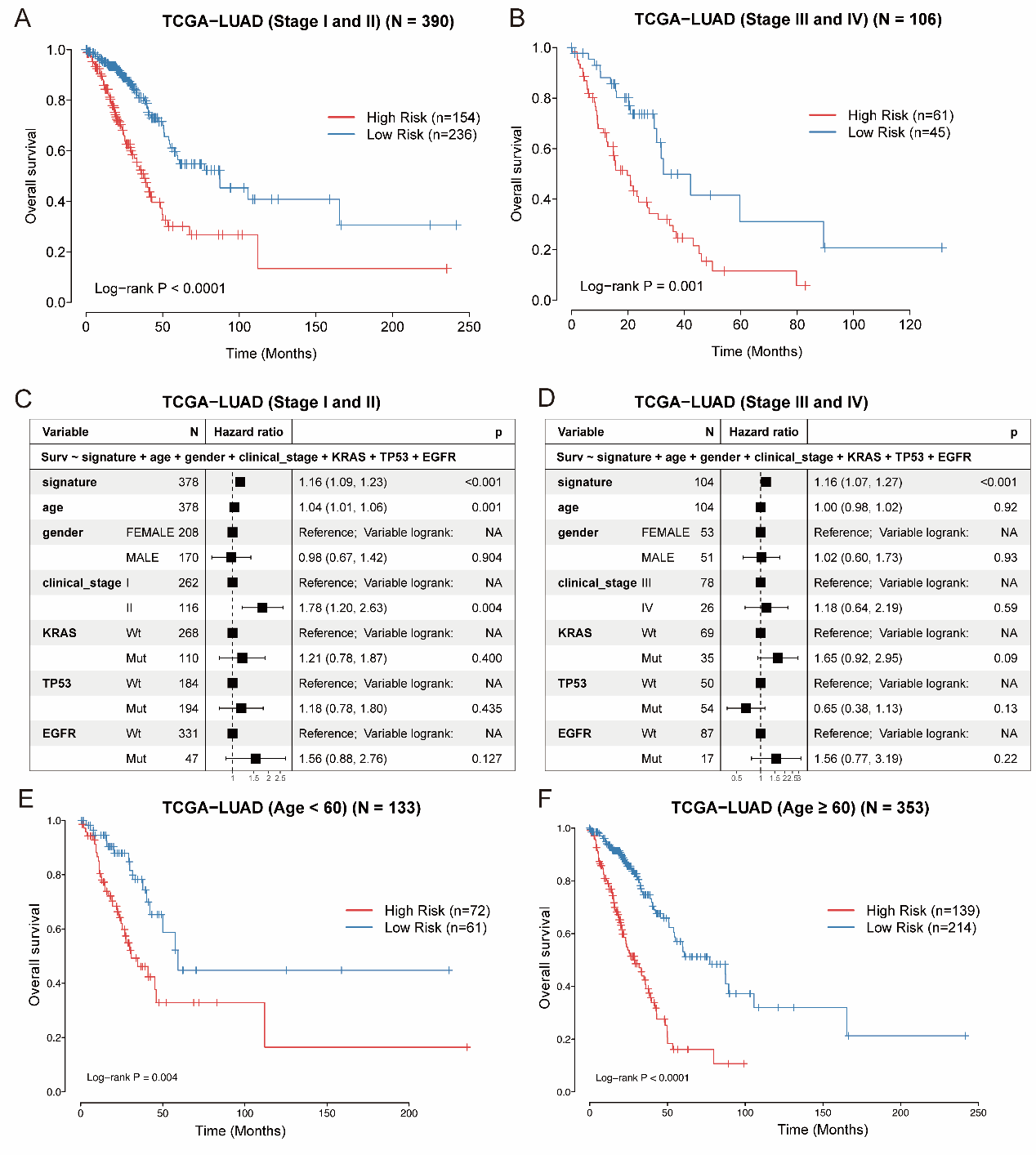


**Kaplan-Meier survival curves or multivariate Cox regression analysis stratified by stage.** Kaplan-Meier survival curves show overall survival for patients grouped as high-and low-risk subgroups in the TCGA-LUAD dataset for the early stage (a) or advanced stage patients (b). Multivariate Cox regression analysis for overall survival in the TCGA-LUAD dataset for the early stage (c) or advanced stage patients(d). Kaplan-Meier survival curves show overall survival for patients grouped as high-and low-risk subgroups in the TCGA-LUAD dataset in patients < 60 (e) or patients ≥ 60 (f).

Figure S4


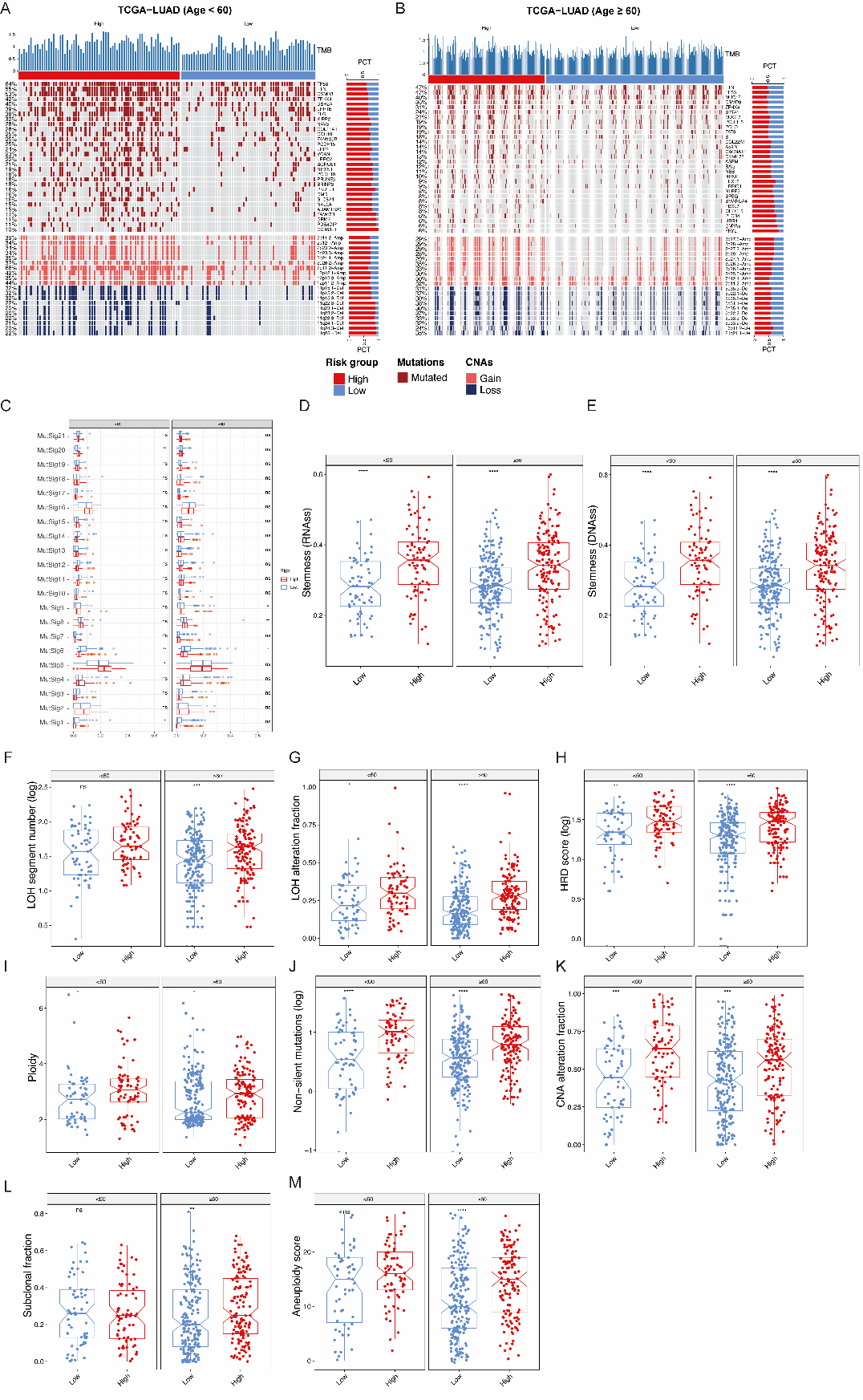


**Characterization of genome alterations in LPRI subgroups stratified by age.** (a-b) Oncoprint shows the differentially altered gene mutations or CNV regions between high and low-risk subgroups in different age groups. (c) Difference in mutational signatures between high and low-risk subgroups in different age groups. (d-m) Comparisons of the stemness, loss of heterozygosity, genome alterations, and instability between low- and high-risk subgroups in different age groups. ‘ns’ means no statistical significance, ∗ means P < 0.05, ∗∗ means P < 0.01, ∗∗∗ means P < 0.001 and ∗∗∗∗ means P < 0.0001.

Figure S5


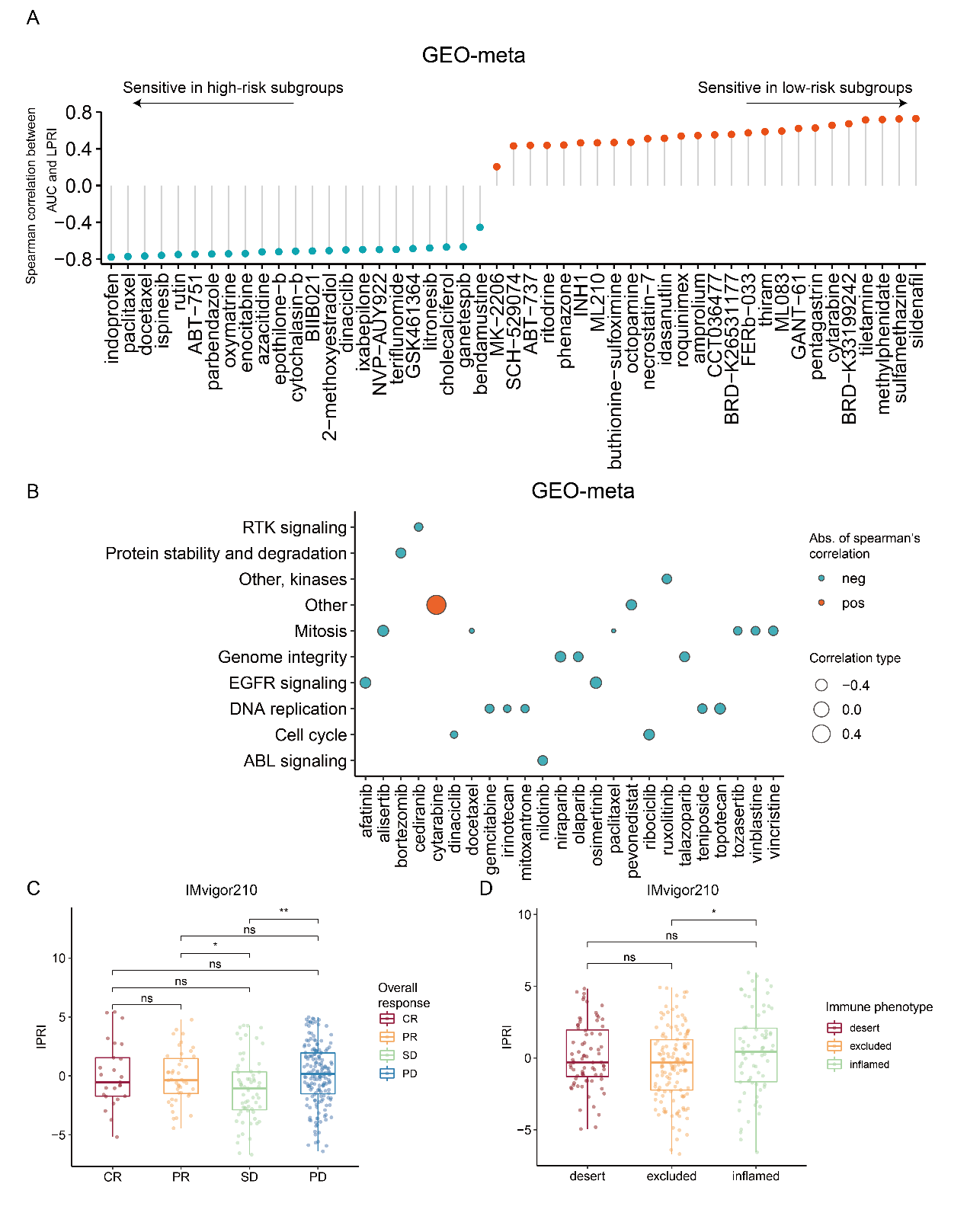


**LPRI predict drug response in the chemotherapy and immunotherapy in the GEO-meta data set.** (a) Spearman’s correlation between LPRI and predicted AUCs of drugs. (b) Pathway annotation of potential sensitive drugs. (c) Difference of LPRI among overall response groups in the IMvigor210 cohort. SD, stable disease; PD, progressive disease; CR, complete response; PR, partial response. (d) Difference of LPRI among immune phenotype groups in the IMvigor210 cohort. ‘ns’ means no statistical significance, ∗ means P < 0.05, ∗∗ means P < 0.01, ∗∗∗ means P < 0.001 and ∗∗∗∗ means P < 0.0001.

**Supplementary Tables**

Table S1. Basic information of included datasets

Table S2. Identification of prognostic regulons

Table S3. LPRI of all included datasets
